# Effect of Oral Pathogens Associated With Pulpitis and Apical Periodontitis on Odontogenic Mesenchymal Stem Cells

**DOI:** 10.1155/sci/5523197

**Published:** 2025-08-31

**Authors:** Linlong Qi, Xiaoyao Liang, Zirui Qin, Huihui Gao, Yi Zhang, Yuan Wang, Shuli Deng

**Affiliations:** Stomatology Hospital, School of Stomatology, Zhejiang University School of Medicine, Zhejiang Provincial Clinical Research Center for Oral Diseases, Key Laboratory of Oral Biomedical Research of Zhejiang Province, Cancer Center of Zhejiang University, Engineering Research Center of Oral Biomaterials and Devices of Zhejiang Province, Hangzhou 310000, China

**Keywords:** inflammatory factor, mesenchymal stem cells, oral pathogens, pulpitis and apical periodontitis, virulence

## Abstract

Dental mesenchymal stem cells (MSCs) play an essential role in the development of immature permanent teeth. Bacterial infection of the pulp and periapical tissues of immature permanent teeth, the associated oral pathogens, and their virulence factors affect the viability, proliferation, differentiation, and cytokine secretion of MSCs. Bacteria and virulence factors can also trigger an inflammatory response that induces pro-inflammatory cytokine secretion and destroys odontogenic MSCs in the pulp and periapical region, negatively affecting the development of immature permanent teeth. The present study explored the role and mechanisms of oral pathogens associated with pulpitis and apical periodontitis and their virulence factors concerning odontogenic MSCs. The findings can contribute to the clinical treatment of pulpitis and apical periodontitis of immature permanent teeth, providing a theoretical basis for improving its clinical efficacy.

## 1. Introduction

Approximately 700 microbial species colonize the human oral cavity, establishing it as the second most complex microbiome in the body, surpassed only by the large intestine. This diverse oral microbiome comprises bacteria, fungi, and viruses [1]. Pulpitis is a common oral disease associated with dental pulp inflammation, with dental caries, trauma, dentin fissures, and dentinal tubules as important pathways for microorganisms' invasion and dental pulp infection [2]. Apical periodontitis is an inflammatory response associated with root canal pathogens and their toxins [3]. Bacteria are the most prevalent and predominant microorganisms causing endodontic and periapical infections [4].

**Figure 1:**
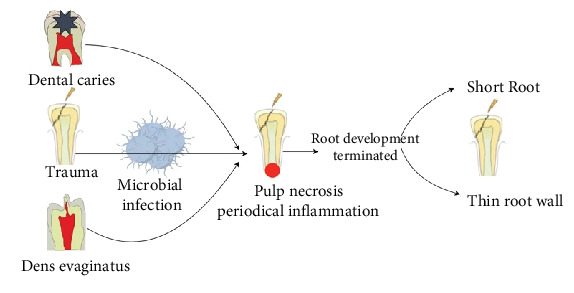
Development of pulp necrosis and apical periodontitis in immature permanent teeth. Dental trauma, caries, and developmental malformations, such as malformed central tips, may cause pulp necrosis and apical periodontitis of immature permanent teeth, resulting in the stop of root development and making the affected teeth with short roots and thin root canal walls.

Upon eruption, immature permanent teeth possess underdeveloped roots, a maturation process requiring 3–5 years for completion [5]. As illustrated in Figure 1, during this critical developmental period, dental trauma, caries, or developmental malformations (e.g., malformed central cusps, dens invaginatus) can precipitate pulpal necrosis and periapical lesions. These pathological processes arrest root formation, leaving affected teeth structurally compromised and prone to fracture or loss. Consequently, this impairs maxillofacial development [6].

**Figure 2:**
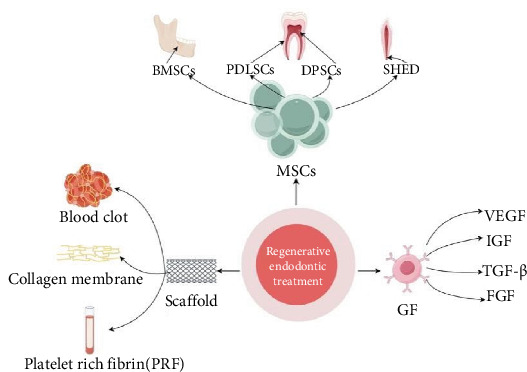
Important factors in pulp regeneration therapy. This figure illustrates the important factors of pulp regeneration therapy, including the following three aspects: (I) MSCs such as bone mesenchymal stem cells (BMSCs), periodontal ligament stem cells (PDLSCs), dental pulp stem cells (DPSCs), and stem cells from human exfoliated deciduous teeth (SHED), are responsible for regenerating pulp-like tissue and hard tissue repair. (II) Scaffold, such as blood clot, collagen membrane, and platelet-rich fibrin, are three-dimensional supporting structures that provide MSCs with an environment for adhesion, proliferation, and differentiation. (III) Growth factors such as vascular endothelial growth factor (VEGF), insulin-like growth factor (IGF), transforming growth factor-β (TGF-β), and fibroblast growth factor (FGF) promote the proliferation and differentiation of MSCs.

Bacteria are the most prevalent and predominant microorganisms causing endodontic and periapical infections [4]. RET aims to regenerate the pulp–dentin complex and associated tissues in these teeth, where root development has been arrested due to pulpal necrosis. The process involves biological and tissue engineering techniques to restore pulpal function, the continued development of roots, the structural reconstruction of cementum and dentin, and the formation of the pulp–dentin complex [7]. The core components essential for successful RET are mesenchymal stem cells (MSCs), growth factors (GFs), and scaffold [8] (Figure 2).

MSCs are one of the elements that promote root development and are involved in forming roots, dentin, pulp, and supporting tissues [9]. MSCs associated with immature permanent teeth mainly include dental pulp stem cells (DPSCs), stem cells from human exfoliated deciduous teeth (SHED), bone MSCs (BMSCs), stem cells from the apical papilla (SCAPs), dental follicle stem cells (DFSCs), stem cells from the pulp periodontal ligament (PPSL) and Hertwig's epithelial root sheath (HERS) [10]. Endodontic and periapical infections are primarily induced by bacteria, which trigger a cascade of host immune responses. Pathogenic bacteria directly invade tissues, release virulence factors, and stimulate the production of diverse inflammatory cytokines. Cell-mediated immunity plays a critical role in this process. Activated inflammatory cells secrete mediators that drive the destruction of periapical tissues [11]. The viability and function of MSCs within critical niches, specifically the apical papilla, dental follicle, and HERS, are significantly impaired by the severity of pulpal/periapical infection, the source of inflammation, and its duration. Severe damage to the apical papilla or dental follicle can compromise the differentiation potential of odontogenic MSCs and subsequent dentin formation, ultimately disrupting root development [10].

This study explores the role of oral pathogens associated with endodontitis and apical periodontitis, their virulence factors, and immune responses on odontogenic MSCs and their correlation with the development of radicular tissues to determine the effects of oral core microorganisms in the root canals of teeth with endodontic and apical periodontitis on root development and speculate on the possible causative mechanisms.

## 2. Dominant Oral Pathogens in the Root Canals of Teeth With Pulpitis and Apical Periodontitis and Their Effect on Odontogenic MSCs

More than 500 microorganisms, mostly bacteria, have been identified in various types of pulpitis and periapical infections, with the main pathogenic microorganisms coming from four phyla: *Firmicutes*, *Bacteroidetes*, *Fusobacteria*, and *Actinobacteria* [12].

### 2.1. Enterococcus

*Enterococcus*, a Gram-positive, facultative anaerobic bacterium, belongs to the *Firmicutes* phylum. *Enterococcus faecalis* is resistant to many drugs and can cause refractory infections and reinfections in the root canal system, making it difficult to eradicate it from the root canal system, even with strict root canal disinfection measures [13]. *Enterococcus faecalis* secretes virulence factors, such as lytic enzymes, gelatinases, hyaluronidase, and cytolysins that cause tissue damage or modulate the immune response of pulp cells, further damaging pulp tissue. Furthermore, *E. faecalis* inhibits cell proliferation and cytokinesis in SCAPs, and transcriptomic analysis reveals *E. faecalis* significantly upregulates VEGFA, RUNX2, and TBX3 expression in SCAPs, suppressing their osteogenic and odontogenic differentiation potential [14].

### 2.2. Porphyromonas

*Porphyromonas*, a genus of Gram-negative anaerobic bacteria within the *Bacteroidetes* phylum, includes *Porphyromonas gingivalis*, a key pathogen implicated in chronic periodontitis development and progression [15]. *Porphyromonas gingivalis* can invade the dental pulp through deep periodontal pockets, potentially inducing retrograde pulpal infection. Exposure to *P. gingivalis* influences the behavior of dental stem cells, while it may promote the differentiation of SCAPs toward mineralization-associated lineages, DPSCs appear to resist its invasion, potentially through regulation of the TGF-β/SMAD, NF-κB, and MAPK/ERK pathways [16]. *Porphyromonas gingivalis* and its lipopolysaccharides (LPSs) exert significant effects on pulp cells. Its LPS reduced alkaline phosphatase (ALP) activity and bone salivary protein (BSP) expression in DPSCs in vitro [17]. Furthermore, comparative studies reveal that both *P. gingivalis* LPS and *E. coli* LPS stimulated inflammatory responses in DPSCs through TLR4 upregulation; however, *P. gingivalis* LPS induced a stronger pro-inflammatory cytokine gene expression profile within the first 24 h post-exposure [18]. It has been demonstrated that after treatment with high doses of *P. gingivalis* LPS, DFSCs have a significantly higher migratory potential and can sense bacterial components but may not play an active role in initiating host immune responses [19].

### 2.3. Prevotella

*Prevotella*, a Gram-negative specialized anaerobic bacterium from the *Bacteroidetes* phylum, has various virulence factors, including adhesins, bacterial fimbriae, hemolysins, proteases, nucleases, and LPSs. These virulence factors may enable *Prevotella* to participate in the progression of inflammatory diseases, such as periodontitis [20]. It was demonstrated that *Prevotella nigrescens* LPS inhibited the differentiation of bone marrow mononuclear cells cultured in the presence of M-CSF and RANKL into osteoblasts but promoted osteoblast formation by decreasing osteoprotegerin (OPG) concentration and increasing TGF-β and PGE2 concentrations in the coculture system [21]. Furthermore, *P. intermedia* LPS significantly enhances NO and IL-1β release from mouse macrophages compared to unstimulated cells [22]. Macrophages can be polarized into two main phenotypes: a pro-inflammatory M1 or an anti-inflammatory M2. M1 cells are mainly induced to produce pro-inflammatory factors, including NO and IL-1β, which promote inflammation and tissue destruction when stimulated by LPS [23].

### 2.4. Fusobacterium

*Fusobacterium*, a Gram-negative anaerobic bacterium from the *Fusobacteria* phylum, is a critical species in biofilm formation. *Fusobacterium nucleatum* bridges primary colonizers (e.g., *Streptococcus* spp.) with secondary colonizers (e.g., *P. gingivalis*) via the adhesins RadD, Fap2, and FomA. It provides a hypoxic microenvironment in root canals to protect secondary colonizers such as *P. gingivalis* [24]. *Fusobacterium nucleatum* is commonly found in oral and systemic infections and is strongly associated with periodontitis, pulp infections, inflammatory bowel disease, and colorectal cancer [25]. One study examined the effect of *F. nucleatum* and its bacterial metabolite-rich supernatant on the osteogenic and odontogenic potential of SCAPs in vitro by RNA-seq transcriptome analysis, indicating that it upregulated the immune and inflammatory responses of SCAPs. It also downregulated WDR5 and TBX2 and upregulated TBX3 and NFIL3 in SCAPs. Upregulation of these genes might be detrimental to the differentiation potential of SCAPs [14]. Under the influence of *F. nucleatum* (bacteria and supernatant), SCAPs produce the pro-inflammatory cytokines IL-6, IL-8, and MCP-1, which has been confirmed at the mRNA level [26]. In addition, *F. nucleatum* inhibited PDLSC proliferation while inducing dose-dependent apoptosis, ferroptosis, and cytokine production [27]. Mechanistically, *F. nucleatum* subverted the STING pathway. *F. nucleatum* initiated autophagy-mediated STING degradation for immune evasion during early infection, then drove STING-dependent IFN-β secretion to amplify endodontic inflammation in later stages [28].

### 2.5. Actinomyces

*Actinomyces*, a Gram-positive, facultative anaerobic bacterium commonly found in the gastrointestinal tract, is an early colonizer of oral biofilms [29]. It is associated with failed endodontic treatments and is usually found in persistently infected extracanalicular lesions [30, 31]. *Actinomycetes* can adapt to oxygen stress, supporting aerobic and anaerobic invasion during the early stages and mutually beneficial coexistence through metabolic communities in the later stages. In vivo bacterial–bacterial recognition between oral *actinomycetes* and *streptococci* is widespread, and their isolates usually show a pattern of coaggregation. Both *streptococci* and *actinomycetes* produce acid from dietary sugars. They are commonly found in carious lesions, and nitrogen compounds in the oral cavity promote acid production by *streptococci* and *actinomycetes* in vivo [32]. When biofilms composed of *Actinobacillus nei*, *Microcystis aeruginosa*, and *Clostridium nucleatum* adhere to the dentinal wall, the residual bacterial biofilm significantly reduces the release of TGF-β1 [33]. The ability of SCAPs to proliferate, differentiate into dentin, and mineralize was significantly inhibited when SCAPs were coinfected with a mixture of oral *Streptococcus* and *Actinomyces naeslundii* [34].

## 3. Effect of Common Virulence Factors of Oral Pathogens on Odontogenic MSCs

Most pulpitis and periapical inflammation cases are caused by the progression of dental caries, in which bacteria and their virulence factors diffuse through the dentinal tubules to reach the pulp cavity, resulting in pulpal inflammation. Common virulence products of oral pathogens include LPS, adhesins, and proteases.

### 3.1. LPS

Gram-positive and Gram-negative bacteria often trigger immune responses via bacterial surface components, such as lipophosphatidic acid (LTA) or LPS [35]. LPS, composed of lipids and polysaccharides, is a significant component of Gram-negative bacteria that induces cellular inflammation [2]. It can activate various downstream signaling pathways by binding to cellular TLRs, leading to the synthesis and release of inflammatory mediators, such as interleukin (IL)-1β, tumor necrosis factor-α (TNF-α), IL-6, IL-8, and cyclooxygenase-2 (COX-2) [36]. As a critical initiator in the pathogenesis of pulpitis, bacterial LPS penetrates the affected pulp tissue and stimulates a massive release of inflammatory mediators in the pulp, triggering a pulpal inflammatory response [37].

LPS is often thought to affect cell proliferation, and it was shown that LPS induces the proliferation of DPSCs in the microenvironment, as well as gene and protein expression profiles. After exposure to LPS, the survival of DPSCs decreased significantly with increasing LPS concentration. Inflammatory factors also increased significantly, and LPS concentrations of 1–2 μg/mL induced inflammation in DPSCs, mimicking the inflammatory microenvironment of the dental pulp in clinical practice [38]. Specific concentrations of LPS can promote cell adhesion and migration of DPSCs by upregulating adhesion molecule and chemokine expression via the NF-κB and MAPK signaling pathways [39]. However, it has been suggested that the effect of LPS on cell proliferation depends on the cell type and the source and concentration of LPS. It has been shown that 5 μg/mL LPS did not significantly affect the proliferation and mineralization of SCAPs [40], and 1 μg/mL *E. coli*-derived LPS did not affect the proliferation, viability, and cell cycle of periodontal membrane stem cells [41], in contrast to a 10 μg/mL concentration of *P. gingivalis*-derived LPS, which enhanced the proliferation of PDLSCs [42].

LPS affects the differentiation of odontogenic MSCs. A previous study showed that LPS did not affect the immunophenotype, proliferation, viability, and cell cycle of PDLSCs. However, it inhibited the osteogenic differentiation of PDLSCs by downregulating the expression of Runx2, ALP, and Ocn mRNAs and stimulated chondrogenesis and adipogenesis in PDLSCs by upregulating the expression of Sox9 and PPARγ mRNAs [43]. Another study found that LPS promoted the expression of inflammatory factors IL-1β, TNF-α, and NF-κB P65 by upregulating the TLR4 signaling pathway. It inhibited osteogenic differentiation of PDLSCs but promoted adipogenesis in PDLSCs. Conversely, appropriate concentrations of LPS stimulate proliferation and osteo/odontogenic differentiation in SCAPs through ERK and p38 MAPK pathway activation [44].

Increasing evidence indicates that inflammatory responses can induce senescence in MSCs [45–47]. Mimicking the inflammatory microenvironment with LPS stimulation promotes senescence in DPSCs. Senescence plays a physiological role in the human body and can also play a pathological role in MSCs, characterized by decreased proliferation, differentiation capacity, and dysfunction. Therefore, the senescence of DPSCs is one of the significant challenges for tissue regeneration therapies. One study has found that LPS from *P. gingivalis* and *E. coli* significantly upregulates senescence-associated genes in DPSCs, including TP53, CDKN1A, CDKN2A, and SIRT1 [48].

### 3.2. Adhesins

There are two types of adhesins: fimbriae and nonfimbriae adhesins. *Actinomyces* type I fimbriae are used to adhere to the tooth or mucosal cell surfaces and mediate adhesion. In contrast, type II fimbriae recognize glycosyl receptors expressed by other bacteria in the copolymer body and mediate aggregation and transcellular interactions [49]. FadA in *F. nucleatum* is a unique bacterial adhesion/invasion hormone that serves as a scaffold for biofilm formation and confers acid resistance to bacteria [50]. FadA acts as a pro-inflammatory virulence factor and can increase the expression of IL-1β, IL-6, and IL-8. Further studies have shown that FadA can bind to PEBP1, activating the Raf1-MAPK and IKK-NF-κB signaling pathways [21]. FomA, another *F. nucleatum* adhesin, may be involved in promoting biofilm formation [51]. Through adhesins, *F. nucleatum* can mediate coaggregation between a wide range of bacteria and plays an essential role in dental plaque formation [52]. LTA is present on the surface of Gram-positive bacteria (e.g., *Enterococcus faecalis*) and is a bacterial surface-associated adhesion factor and a regulator of cell wall autolysins [53]. It has been demonstrated that DPSC expresses TLR2 and activates the NF-κB pathway in response to LTA stimulation. LTA stimulates DPSC proliferation, affects the adhesion capacity of the cells, accelerates cell migration, and induces IL-6/IL-8 secretion but does not regulate osteogenic differentiation [54].

### 3.3. Protease

Bacterial proteases are important virulence factors for the development of infections. Bacterial protein hydrolysis activity gives rise to the degradation of connective tissue and the hydrolysis of proteins involved in host defense mechanisms [55]. Protein hydrolases, including cysteine and serine proteases produced by *Prevotella* spp., contribute to the destruction of periodontal tissues by breaking down cellular peptides and degrading the collagen matrix in periodontal tissues [56]. *P. intermedia* requires hemoglobin for growth, survival, and virulence, which it obtains through the protein hydrolase interpain A or albuminase, which degrades hemoglobin in inflamed periodontal pockets under specific conditions of low redox potential and high pH [57]. *P. gingivalis* produces a unique group of cysteine proteases called gingipains, including Arg-gingipain (Rgp) and Lys-gingipain (Kgp). Rgp and Kgp together promote *P. gingivalis*-induced cell migration and pro-inflammatory mediators by activating protease-activated receptor 2 cell migration and expression of pro-inflammatory mediators [58]. *P. gingivalis* has multiple effects on the host cells' immune response, including cleavage of T cell receptors, such as CD2, CD4, and CD8, inhibiting the cellular immune response, and stimulation of the expression of protease-activated receptors on neutrophils, which releases pro-inflammatory factors and enhances inflammatory responses [59].

## 4. Mechanisms of Oral Pathogens and Their Virulence Factors Affecting the Proliferation and Differentiation of Odontogenic MSCs

As explained above, bacterial virulence factors such as LPS induce inflammation and the production of pro-inflammatory cytokines in odontogenic MSCs. Inflammation is a cellular defense mechanism against foreign agents, but a sustained inflammatory response can lead to some pathological changes [60]. Pro-inflammatory cells and cytokines in periapical tissues trigger inflammation and damage periapical tissues. According to previous studies, the main inflammatory factors measured in the exudate of pulpitis and periapical inflammation in immature permanent teeth are ILs, TNF, interferon (IFN), and transforming GF (TGF) [61–64], which regulate the host immune response and form a complex regulatory network that affects root development in immature permanent teeth by activating or inhibiting osteoclasts.

### 4.1. ILs

ILs transmit messages, activate and regulate immune cells, mediate T and B cells' activation, proliferation, and differentiation, and play an important role in inflammatory responses. Studies have shown that different ILs synergize or constrain each other, constituting a complex immunoregulatory network for regulating various physiological and pathological responses in the body. ILs include IL-1, IL-2, chemokines, IL-6, IL-10, and IL-17 families.

IL-1 is an important pro-inflammatory cytokine secreted by monocytes, T cells, dendritic cells, and macrophages. According to the literature, IL-1 expression is significantly elevated in diabetic rats with periodontitis, and IL-1β promotes osteoclast formation in patients with rheumatoid arthritis, periodontal disease, and osteoporosis [65–67]. IL-1 plays an important role in osteoblast signaling for osteoclast formation. It has been reported that inflammatory factors affect the differentiation ability of DPSCs, and osteogenic differentiation, chondrogenic differentiation, and lipogenic differentiation of DPSCs are significantly inhibited under the stimulation of IL-1β [68].

IL-6, a significant mediator of the host response to tissue injury and infection, induces osteoclast differentiation by upregulating adhesion molecules that induce angiogenesis and increase vascular permeability and inflammatory edema [69]. IL-6 promotes osteoclast activity for bone resorption by inducing RANKL expression [70] and contributes to bone resorption in diseases such as periodontitis, rheumatoid arthritis, and osteoporosis; it is also a promoter of bone resorption by osteoclasts. DPSCs are regulated under inflammatory conditions to release IL-6, one of the key molecules in pulpal inflammation; IL-6 can also inhibit neurulation while promoting osteogenesis [71].

IL-8 is an important cytokine in human dental pulp tissues, which chemotactically recruits neutrophilic leukocytes, basophils, T-lymphocytes, and monocytes. It also induces the expression of adhesion molecules, traverses the vascular endothelium, and mediates inflammatory responses. Studies have shown low concentrations of IL-8 expression in normal pulp tissue exudate, with significantly increased levels in the inflamed pulp tissue [72]. IL-8 upregulates RANKL expression in osteoblasts and directly induces osteoclastogenesis by binding to supracellular CXCR1, stimulating osteoclast differentiation and bone resorption [73]. In addition, IL-8 synergizes with other inflammatory factors to form a complex network to regulate osteoclast differentiation. For example, IL-8 stimulates the upregulation of TNF-α, IL-1, and IL-6, collaboratively promoting bone resorption [74].

IL-17, a pro-inflammatory cytokine secreted by a newly discovered subpopulation of cells, has a potent recruitment function for neutrophils and stimulates the secretion of various inflammatory mediators by epithelial cells, endothelial cells, fibroblasts, osteoclasts, and macrophages, among others [75]. Some researchers dynamically explored periapical inflammation and alveolar bone destruction by establishing a rat model of apical periodontitis. The results showed that with the development of apical lesions, the expression of IL-17-positive cells increased, mainly concentrated around the abscess, suggesting that IL-17 contributes to the development of apical periapical inflammation and may promote osteoclast differentiation [76]. It has been demonstrated that IL-17 induces the proliferation and clonogenic capacity of SHED. It can also promote early osteogenesis of SHED and DPSCs by increasing ALP activity but does not significantly affect late osteogenesis [77].

On the other hand, IL-10 and IL-27 inhibit the secretion of inflammatory factors, such as TNF-α, IL-1β, and IL-6 [78]. IL-10 is an important anti-inflammatory cytokine. It can inhibit osteoclast differentiation through direct action on osteoclast precursors. One study constructed a mouse periodontitis model; compared with the control mice, the area of bone resorption in the alveolar bone of mice injected with IL-10 was reduced, suggesting that IL-10 is involved in inhibiting bone resorption [79]. IL-27 is produced by antigen-presenting cells and has a pleiotropic immune function. According to some studies, IL-27 inhibits the differentiation of mouse osteoclasts. In addition, IL-27 inhibits RANKL-activated ERK, p38, and NF-κB signaling by downregulating the expression of RANK and TREM-2, effectively inhibiting osteoclastogenesis and osteoblast activity [80].

IL-37, an anti-inflammatory cytokine, is a member of the IL-1 family. Unlike other members, it inhibits the cellular production of pro-inflammatory cytokines and has anti-inflammatory effects [81]. IL-37 can inhibit inflammatory processes through the intracellular SMAD3 and extracellular IL-18Rα signaling pathways [82]. IL-37 has been reported to promote osteogenic differentiation of human bone marrow MSCs by activating the PI3K/AKT signaling pathway [83]. According to another study, IL-37 can activate autophagy in DPSCs, which enhances the osteogenic and odontogenic differentiation of DPSCs, while having no significant effect on proliferation [84].

### 4.2. TNF-α

TNF-α is also an inflammatory factor that stimulates monocytes, activates macrophages and T cells, and induces their synthesis of inflammatory factors such as IL-8. Significantly higher serum levels of TNF-α have been reported in patients with acute pulpitis. After treatment, serum levels of TNF-α decreased considerably, suggesting that TNF-α may be an important inflammatory mediator in endodontic infections [85]. It has also been reported that TNF-α in the inflammatory exudate from periodontal pockets of patients with periodontitis can induce osteoclastogenesis and promote alveolar bone resorption by activating the NF-κB pathway [86]. Several in vitro studies have shown that TNF-α activates the NF-κB pathway and promotes osteogenic differentiation of DPSCs [87, 88]. However, short-term exposure to TNF-α (6 and 12 h) induces apoptosis of DPSCs by inducing the NF-κB pathway. In contrast, long-term exposure (14 days) promotes the proliferation of DPSCs and may inhibit the mineralizing potential of DPSCs. An in vitro study showed that TNF-α increased the migratory activity of DPSCs [89].

### 4.3. IFN

IFN family members include type I IFNs (IFN-a and IFN-b) and IFN-c. IFN is a critical cytokine in the immune response. It triggers the production and release of reactive oxygen species from macrophages and is associated with periapical immune responses. Some researchers have compared IFN-γ levels between normal and inflamed pulp tissues; its expression significantly increased in endodontitis. IFN can inhibit osteoclast differentiation through a RANKL-mediated negative feedback loop, suggesting that IFN inhibits bone resorption [90]. IFN-γ has been reported to improve impaired dentin formation and immunosuppressive function of pulp stem cells derived from an irreversibly inflamed pulp [91]. According to a previous study, high concentrations of IFN-γ did not significantly affect the proliferation of DPSCs [92]. In contrast, low concentrations of IFN-γ could promote the proliferation and migration of DPSCs, in addition to the fact that low concentrations of IFN-*γ* inhibited the odontogenic and osteogenic differentiation of DPSCs through the NF-κB (p65) and MAPK (P38) pathways [93].

### 4.4. TGF

The TGF family encompasses a group of proteins that regulate cell growth and differentiation, with three isoforms in mammals: TGF-β1, TGF-β2, and TGF-β3 [94]. TGF-β exerts a dual regulatory effect on osteoclast differentiation, generation, and function [95]. It can block the inhibitory signals of osteoclast formation and promote osteoclastogenesis by upregulating osteoclast cytokine signaling inhibitory proteins. In addition, it can also upregulate the ap-1 family member jum-b to promote osteoclastogenesis. TGF-β1 has been reported to enhance the viability and osteogenic differentiation of DPSCs through the MAPK signaling pathway, which promotes the expression of osteogenesis-related genes in DPSCs [96]. TGF-β1 can also regulate the growth, collagen deposition, and differentiation of SCAP and promote repair during activation/hematopoietic remodeling for new tissue formation by upregulating PAI-1 and downregulating uPA [97].

## 5. Conclusion

The roots are not fully developed when permanent teeth erupt. Odontogenic MSCs play a significant role in root development. Trauma, caries, and developmental anomalies in immature permanent teeth can lead to inflammation of the pulp and periapical region, affecting root development [98]. Oral pathogens play a crucial role in this disease process. Currently, in immature permanent teeth affected by pulpitis or apical periodontitis, the affected tooth can be preserved by pulp regeneration therapy to heal the periapical tissues and promote the continued development of the root of the affected tooth. However, at this stage, the affected tooth is structurally characterized by a short root, a weak root canal wall, and an open apical foramen. Therefore, mechanical debridement is not recommended for pulp regeneration therapy, which may ultimately lead to treatment failure if root canal disinfectants cannot effectively control bacterial infection in the root canal [99, 100]. It is crucial to become knowledgeable about the oral pathogenic microorganisms in the root canals of immature permanent teeth with pulpitis and periapical inflammation and understand their relationship to pulp regeneration in immature permanent teeth.

**Figure 3:**
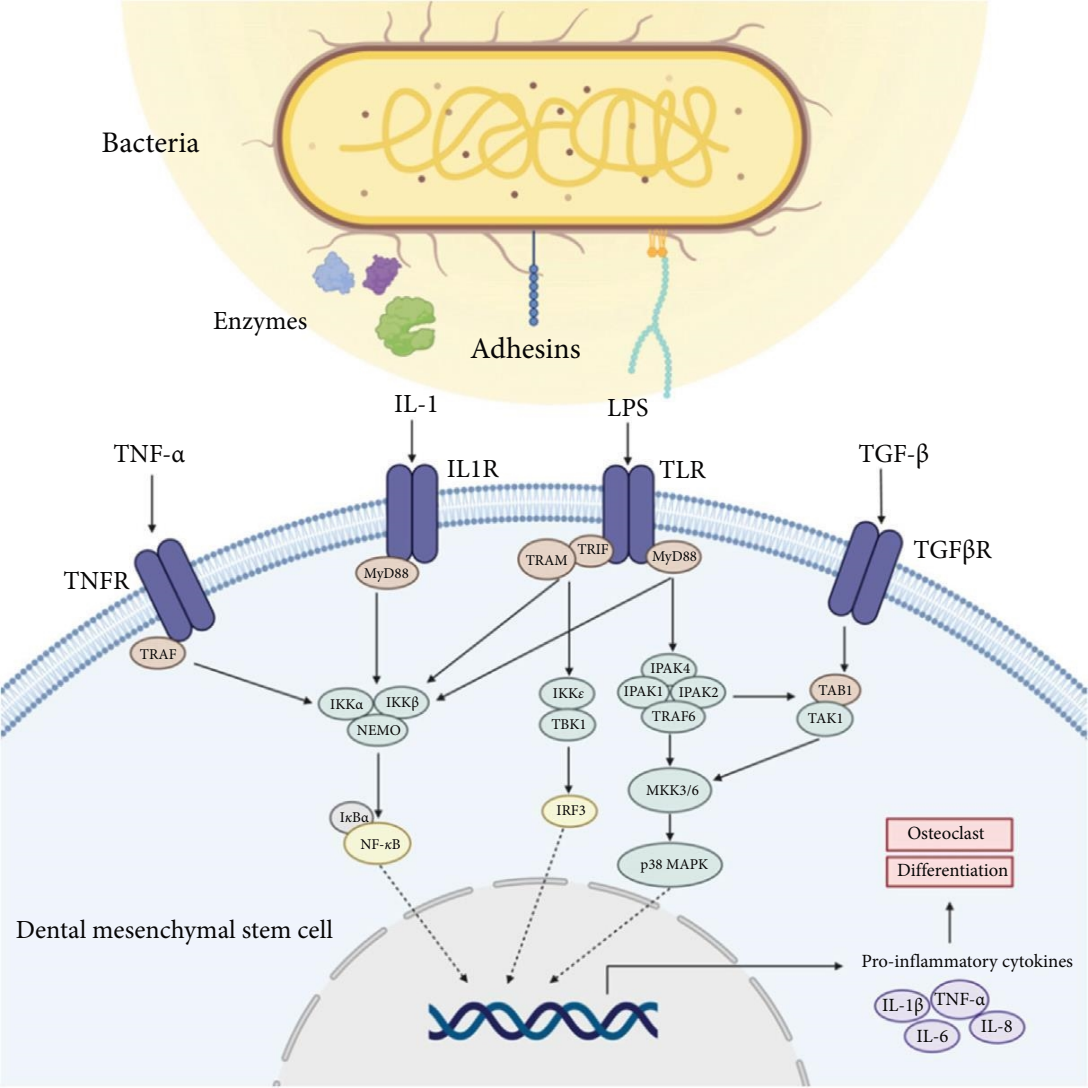
Mechanism of oral pathogens associated with pulpitis and apical periodontitis on odontogenic mesenchymal stem cells. Oral pathogens induce tissue damage and modulate the immune response of odontogenic stem cells through virulence factors (e.g., adhesins, proteases, and LPS), compromising stem cell viability. In pulpitis and apical periodontitis of immature permanent teeth, inflammatory mediators such as ILs and TNF activate the NF-κB (p65) and MAPK (p38) signaling pathways. This triggers the release of pro-inflammatory cytokines, which subsequently impair the osteogenic differentiation capacity of MSCs, ultimately disrupting the development of immature permanent teeth.

Most of the dominant oral pathogens in the root canals of teeth with pulpitis or apical periodontitis were from the phyla *Firmicutes*, *Bacteroidetes*, *Fusobacteria*, and *Actinobacteria*, which are predominantly Gram-negative anaerobes. The microbial composition of the root canals of immature permanent teeth with pulpitis and apical periodontitis is comparable to that of mature permanent teeth. Oral pathogens can cause tissue damage directly or indirectly through virulence factors, including adhesins, fimbriae, hemolysins, proteases, nucleases, and LPS, or modulate the immune response to odontogenic stem cells, further damaging stem cells. Pro-inflammatory cells and cytokines in periapical tissues initiate inflammation. Inflammatory factors associated with pulpitis and apical periodontitis in immature permanent teeth, such as IL, TNF, and IFN, modulate the host immune response, forming a complex regulatory network that affects the development of the immature permanent teeth by activating or inhibiting MSCs (Figure 3).

This study has several limitations. Much of the supporting evidence is derived from in vitro investigations, while corresponding in vivo data are lacking. Furthermore, the concentrations of pathogens and their virulence factors used in this study may not resemble those encountered in vivo. Additionally, the presence of endogenous anti-inflammatory factors in vivo, along with complex microbial interactions, could potentially alter the biological behavior of odontogenic MSCs—including their inflammatory responses and differentiation potential.

Nevertheless, this work offers valuable insights for informing clinical strategies aimed at preserving the vitality of immature permanent teeth affected by pulpitis or apical periodontitis. Specifically, it underscores that rigorous disinfection protocols are essential during pulp regeneration therapy. Whether employing irrigation with sodium hypochlorite (NaOCl) and chlorhexidine or adjunctive antimicrobial agents, these interventions exert significant bactericidal effects against pathogens and biofilms within the root canal system.

Future research should prioritize elucidating the pathogenic mechanisms of pulpitis/apical periodontitis-associated pathogens to facilitate the development of more effective disinfection and sterilization approaches, potentially through innovative technologies or optimized combinations of antimicrobial agents. Concurrently, investigations into the interactions and synergies among these pathogens, as well as the identification of pharmacological agents or methodologies capable of mitigating their detrimental effects on odontogenic MSCs, are crucial for enhancing the success rate of pulp regeneration in immature permanent teeth.

## Data Availability

The data that support the findings of this study are available from the corresponding author upon reasonable request.
